# Pediatric Hospital: The Paradigms of Play in Brazil

**DOI:** 10.3390/children2010066

**Published:** 2015-01-29

**Authors:** Lino de Macedo, Gláucia Faria da Silva, Sandra Mutarelli Setúbal

**Affiliations:** 1 Instituto Pensi, Fundação José Luiz Egydio Setúbal, Av. Angélica, 1987, 17o. Andar, 01.227-2000-São Paulo/SP, Brazil; 2 Hospital Infantil Sabará, Fundação José Luiz Egydio Setúbal, Av. Angélica, 1987, 17o. Andar, 01.227-2000-São Paulo/SP, Brazil; E-Mails: glaufaria@live.com.br (G.F.S.); smutarelli@gmail.com (S.M.S.)

**Keywords:** pediatric hospital, humanization, Winnicott, Piaget, play, therapeutic toys, Brazil

## Abstract

The role of play in Brazilian children’s hospitals is highlighted, as well as the perspective of humanization in Brazil. Some aspects of our culture are crucial to understanding the importance of play considering our society. Sabara Children’s Hospital (“Hospital Infantil Sabará”) in Brazil is used particularly to discuss humanization. To understand the issue of play in Brazil, it is important to discuss hospitals in their social context, their history, current roles in children’s care, humanization history and child development, according to the approaches of Piaget and Winnicott that are used in our culture.

## 1. Introduction

Although hospitals are a recent institution in Brazil (less than 100 years), it is a fact that, nowadays, in our country, families use children’s hospitals for childcare. Even more recent are the playrooms inside children’s hospitals. The importance of play was recognized only in 2005, by incorporating the law that obliges each hospital to install a playroom when it has children in-patients.

The presence of a playroom does not mean that play is happening. It will only occur if the hospital has a humanization culture. In Sabará Children’s Hospital, the playroom is fully occupied with sick children exchanging feelings, experiences and ideas, while playing games, or painting, or listening to stories, according to the program designed for that day and hour.

## 2. Results and Discussion

### 2.1. The Hospital: Social Context and the Present Moment

Since the middle of the last century, the fundamental mark of these institutions has been linked to the original meaning of the word, hospital, which comes from the Latin, *hospitium*, the place where visitors were received. With the passing of time and the evolution of cities, the word hospice started to refer to establishments permanently occupied by poor, sick people, the incurable and the insane, and the name, hospital, indicated houses where sick people received temporary treatment.

In the book, *History and Evolution of Hospitals in Brazil* (“História e Evolução dos Hospitais no Brazil”) [1], one can read that the healthcare in our country was precarious. The clinics were meant only for the high social classes, and the hospitals were designed for the poor. There were no trained professionals (nurses, doctors, assistants), and the struggle included a differentiation between medical and social care. There were no clinicians working together as a team. One must consider that there was one nurse for every 37,000 inhabitants. Nowadays, there are nearly 4 nurses for every 500 inhabitants. Only in 1944 did professors from Sao Paulo University reorganize the hospital system into something close to the system we have today, with clinicians working as a team on each a patient.

In recent years, technology has come to permeate our daily lives in an irreversible way. The whole of society has been affected: health, education, manufacturing, financial services and communication. In other words, new technologies allow for and require new ways of interacting with people.

With the difficulties with respect to the social differences in Brazil, the public hospital is a privileged place for the deposition of the symptoms of contemporary disease, which is much more than healthcare itself, but also psychosocial care. Families have been arriving with their demands and anguishes at the hospital. Therefore, one can find in the hospital medical knowledge combined with technology, but also difficulty during the discharging of patients and families. They need to cope with the hospital situation before they go home.

Raising a child, playing with a child and treating a child are really about expending time sharing, being together, looking, seeing and communicating and letting him/her cope with the processes he or she has been experiencing.

### 2.2. Humanization

The notion of comfort became important in 1928 in France. In the 1930s, the concept was extended to well-being. The principle of humanity appeared in 1949. “It is necessary to humanize hospitals” became a slogan [2]. Decades later, the word humanization became obsolete and was replaced by the term, quality. This term became important again in the 1980s and 1990s, with projects on the humanization of care. Nowadays, humanization is one concept used when a hospital is concerned with both health and psychosocial care [2].

Humanization policies entered onto the Brazilian scene in 2000. There was a nationwide push for the Unified Health System (Sistema Único de Saúde), redefining the concept of health as a political and social action, capable of creating not only healthcare, but also psychosocial care [3,4].

It is our interest, therefore, to explore the relationship among clinicians, patients and their families as an open, continuous and essentially relational process. In Brazil, it is common to see entire families become affected to the point of dysfunction by a child’s illness. Families can face illness in a way that can damage their perception of the disease, as well as the sick child’s perception. It should be emphasized that, institutionally, it is possible to support one or the other stance when the whole staff is aware of this [5].

From an ethical perspective, human projects are not accomplished through actions, but are instead realizations of values. Values are neither subjective, nor objective, as they are constructed both individually and collectively. As human constructions, they are constantly changing. There is always a subjective moment that is objectified in an act that is trigged by values. Values are the most important human elements and not recognizing their fundamental role is a tragedy in the practice of medicine [6].

From this perspective, humanizing means not only making every day in hospitals lighter and more playful, but also taking a stance that targets, identifies, makes visible and respects the values of the patient and his or her family [7].

### 2.3. The Child’s Development According to Winnicott

Donald Woods Winnicott (1896–1971) was a psychoanalyst and pediatrician at Paddington Green Children’s Hospital, London, for over 40 years. His perspective on children’s development differs from the classic trends of psychoanalysis in many aspects, keeping, nonetheless, its topicality and influence. We will outline the propositions about the formation of the self, a fundamental axis to think about play as a space for subjectivity, creativity and self-healing [8].

In psychoanalysis, it is known that the self is a structure that is organized little by little, from birth onwards. The baby is born with an inherited potential and characteristics that must be known and respected; for instance, a child might be quiet and sleepy or restless and hungry. The first weeks are difficult. It is the moment when mother and child need to get to know each other, and this familiarity will guide the rhythm of care, of sensations that are permitted or avoided, of body movements, of the tones of affection that prevail at every micro moment of care, made of endless sequences of presence, stimulation and absence.

In Winnicott’s theory, dependence and inter-subjectivity are founding axes of the human psyche. Such inter-subjectivity, however, has strict rules that transpire in the concept of the sufficiently good mother. This is the mother who can modulate the needs of the care of her baby’s self. From the baby’s perspective, the sensorial stimulations, simultaneous or sparse, will form a “mantle” of well-being derived from her or his connection with the care. That (discomfort, cold, hunger, sleepiness) disappears when that other appears (milk, lulling, singing). This modulation will allow the psyche to come out with all its potential and begin the complex unit called the psychosoma.

If the first year of life goes well, the self will come out sustained by the environment that provided the adaptation for his or her maturation process without interruptions. The illusion process will go its course simultaneously with the slow and gradual beginning of frustration, until the initial omnipotence gives way to progressive contact with reality [9]. The union between maturation, care and development will slowly provide moments of gradual differentiation, accompanied by pleasure and anguish.

At this point, it is important to keep the sensation of the continuity of himself or herself and the trust in this continuity, moreover, when noticing, simultaneously: (1) the emergence of the mother as a person who sustains the baby with her body, her looks, her face; and (2) the emergence of himself or herself as a unit, connected and dependent on the mother. The mediation between the baby’s initial omnipotence, the process of illusion and disillusion, which gives way to the real world, will be a task made easier by the transitional object.

The transitional object, therefore, will be the object that can keep the illusion of the continuity of the presence of the baby and parents, and at the same time, this same object will enable gradual and secure detachment of the child from his or her parents. This intermediate area born of the adaptation of the environment, of the trust in intersubjectivity, is called potential space, the space from which play will be born. Play will always be, for Winnicott, in childhood or in adult life, the way to restore trust in one’s self and in the world as we experience it.

### 2.4. Being in the Hospital for a Child

#### 2.4.1. Sickness

Being hospitalized for children is an adverse situation in and of itself. It is something they did not want, and sometimes, it is felt as a tragedy. Hospitalization changes a child’s life routine. They can collaborate, reject or resign, cry, become aggressive, depending on the capability of the clinicians to care for the sick child and of her or his family to cope with the hospital situation. In other words, getting sick and having to go to the hospital can be felt by the child as a threat. This is called the maladaptive cycle of threat, danger, fear, distress, pain, aversion and avoidance [10]. The child is afraid of being abandoned.

#### 2.4.2. Toxic Stress

One of the factors that disturbs the healthy development of a small child, according to the Center on The Developing Child at Harvard University [11], is toxic stress. In a word, this stress refers to the marks left on the child by the adversity of negative experiences and which affect his or her development. It is known that, when a child feels threatened, its body responds by raising its heart rate, blood pressure and stress hormones (cortisol). According to the Center, the intensity, the duration and the repetition of situations that bear these responses may have damaging effects on learning, behavior and health throughout life. It is also known that the presence of adults participating, taking care of or avoiding these occurrences may prevent these damages. In other words, a disease, occasional or chronic, and the ways a child copes with it can cause toxic stress and influence the child’s development negatively. How adults respond in these situations can mitigate the child’s suffering and be favorable for the child’s present and future.

There are two other forms of stress that are not toxic. These can be positive in the sense that every child’s interaction with the external world is supposed to create a certain tension, and the outcome can be positive. If there is an obstacle, problem or interest, it requires an effort or a certain level of excitement for the response, and the cycle is closed. Then, the child can rest and move on to other things. Consequently, there are stresses (most of them) that are positive when they are favorable for the healthy development of the child. There is also tolerable stress, which refers to apprehensive situations, such as the loss of a dear person or the death of an animal. If the child can count on the positive help of an adult and overcomes this situation, the damaging effects are not permanent.

Let us consider that stress that is not positive, tolerable or toxic in and of itself. Rather, it depends on the ways or the sensitivity of the child’s responses to the stimulations or the situations that produce them, as well as the individual perception of certain events and their consequences for the short, medium and long run. Perception is a complex concept, especially in childhood. The child’s perception is deeply connected to its parents’ perception and family narrations. In a children’s hospital, two fields of intervention, which fundamentally connected and different, are opened:

− The perception and the impact of getting sick on parents (time perspective);
− The perception and the impact of getting sick on the child (reverberations).

The perception of children includes their hypotheses about getting sick, the etiology and the evolution. Reality and fantasy interlace, forming a reality according to which the child responds to the world and to treatment. Thus, it is difficult to assert that there are children who are frailer and others who are more resilient to the same stressful occurrence or if the environment’s response contributes to these characteristics. What matters, however, is that the former are the ones that get harmed the most, even more so if certain negative situations that cause suffering, fear or threats occur frequently. In this sense, toxic stress, little by little, produces a pattern of defense, resentment or fear in the face of several things and people, which has a repetitive and cumulative effect, affecting the child’s mental, emotional and physical health for life. It is not by chance that these are the children who, as they become youngsters and adults, may experience health problems, drug abuse, depression and difficulties in succeeding socially, whether it is in school, at work, starting a new family or in their behavior generally as adult citizens [11].

What is applicable to the child is also valid for adults, their parents or guardians. Many of them are a positive reference to the child, because of the care and importance they give to them, because of the love they dedicate to them and that feeds the child’s life and development. Nevertheless, there are others who often behave in a frail manner, commit abuse, are negligent, suffer from mental illness, use drugs and become violent and unable to take care for their child, because, in such cases or moments, they cannot or do not know how to look after themselves. It is not the case to deny them the right or the wish to have children, but to admit that they, the parents and children, need social and institutional support. One has to recognize that children, no matter who their parents are and what they can do for these children, belong to a society, are part of the world and express a moment in the cycle of life, one that needs to be recognized as a right and necessity to receive optimum standards in the possibilities of growth, not for themselves, but for all of us. Children, no matter who their parents are and what they can do for these children, are the future that we, as adults, must care for, protect and offer the best conditions for its existence.

Hence, when we refer to humanization, we will always be referring to play as a tool to help children to cope with hospital procedures that might be traumatic.

### 2.5. Play in the Hospital

“Everything seems very simple when it goes well” [12], says Winnicott. His wisdom overflows the limits of his writing’s intention, allowing us to go beyond the quotation above for play.

“When a woman becomes a mother, she can be a mother in such a natural way, we should never interfere. She will not be able to fight for her rights, because she will not have an understanding of the real world. Everything she will know is that she has been wounded. The only difference is that the wound is not a broken bone or a cut in her arm, but the baby’s mutilated personality. It is common that a mother may spend years of her life trying to heal this wound, when it was actually caused by us when, for no reason, we interfered in something that was so simple that it did not seem to be important at all” [12].

The development of psychism, humanization in its many perspectives, becoming sick, playing or the negative aspect of toxic stress, each phenomenon in its own way reopens the fundamental issue that permeates the construction of this work: intersubjectivity. Whether it is between parents and baby, staff and family, caregivers and child and, above all, in the countless forms of its absence, intersubjectivity does not only sustain and guide the essence of who we are, but participates customarily in the foundation of who we can become.

Back to Winnicott, in the early moments of the most essential intersubjective relation, a delicate area of sharing pleasure and trust gains consistency and sacredness between a mother and her baby. This area is called potential space, and it has the power to overcome one of the main risks of early development: the contact between the internal and the external world [13]. To have an idea of the relevance of this communication, countless severe psychopathological conditions are recognizable from this fracture: paranoia, delusion or hallucination represent, in a way, the overlapping of the internal world over the external one.

If psychopathology exemplifies the fracture, play represents the cure: in each moment a child plays, the possibility and the main tool to appease and weave links between the internal and the external perception, remakes moments of subjective reassurance.

“The essential characteristic of what I wish to communicate refers to play as an experience, always a creative experience, an experience of space-time continuity, a basic form of living” [13].

To Winnicott, there is a direct evolution of the transitional phenomena to play, from play to shared play and from this to cultural experiences. Thus, it can be observed that in the passage from intersubjectivity of the first months, the way toward the first organization as a separate self is paved. Such an experience initiates play as a necessary experience in the creation of this source area for playfulness, relationships and games, as well as the fruition of symbolic and cultural aspects [13].

Piaget considers the ludic relation of the child with objects (the mediators are internal and refer to what the child can and wants to do with them) [14]. For Piaget, as for Winnicott, what is considered as a preparation of the possibility of playing is the extreme dependence of the self on his or her immediate environment, this regarded as the basis for the building of the ego and, consequently, of the object (without the building of the ego, the self does not differentiate as a unit; as a phenomenon, when we observe a child, we see something similar to the symptoms of the autism spectrum).

Observing a small child playing is a fascinating experience. Let us consider a child interacting with objects. If the playing activities involve only the child and objects, it is a play exercise, according to Piaget [15]. These are repeating actions, which she or he knows how to do, for functional pleasure. It is the child who chooses the actions that are better for him or her. The child considers the object’s functions and physical characteristics. What kind actions can we observe in this experience? Children can look at an object, beat, push, fill it up with things and then empty it again, smell, lick, produce sounds, squeeze, hide it from sight and then look at it again, turn around, emit sounds, *etc*. It is the child who, in an absorbed, focused way, chooses what, when and how to do this. This play exercise does not require a particular structure. The functional pleasure, the repetition of what the child wants to repeat, is enough.

It is important, at the hospital, that a child has opportunities to play this way. In this context, we observe that sometimes what pleases a child is drawing or painting a picture and, soon after, painting or drawing another, repeatedly. In these moments, it seems like the best thing for the child is to focus on the things she or he knows and likes to do. The child has the chance to decide what to do, at least in this situation, having some self-control over his or her life and interests.

Nevertheless, we can observe or promote another playful activity that is equally important. In this case, the source of the play, who moves it or promotes it, is an adult, who dances, clowns, sings, tells stories, proposes tasks or activities, starts conversations, laughs, shares something in common or just stays near. In such situations, the initiative belongs to the other, and the child interacts by saying or showing how and how much he or she likes the proposal. In this case, it is the child’s relation with adults that is healing, and the objects or things used are merely a means to link them. Being passive, quiet and ignoring are situations experienced by hospitalized children in our country. If someone comes with a different, interesting proposal, which takes the child from the situation he/she is in, this can be very healing for that child.

At Sabará Children’s Hospital, these two forms of promoting play for the small child are put in practice. There are objects, games and materials that children can choose to play with, relying only on themselves. There are also people—volunteers and hired professionals—who sing, clown, tell stories and carry on interesting activities. There are dogs to be touched and petted, dogs whose patience, presence and tolerance bring peace, joy and comfort to the children. It is important to know how to provide these different forms of ludic experiences. It is important to stimulate and enable, even within the limitations for which the child is in the hospital, so that the child can take initiative, search and decide what she or he knows and wants to do. It is important, also, that adults be present and likewise take initiative, make proposals, take away—in a playful and light manner—the child from that moment and situation she or he is in and lead him or her to another situation.

In other words, there are two situations: children can play with objects and assimilate them by themselves, or they can react, participate, follow or imitate external initiatives, which come from the external world, moreover when these can create or demonstrate nice and funny things to be seen or enjoyed. These two sources—the external, coming from the social aspect, and the internal, from the personal aspect—meet after the child is six or seven years of age, when the symbolic and exercise games become games with rules. In this case, the two aspects—personal and social—become inseparable. In fact, in this game structure, the rules and objectives are social, cultural and anthropological. It is about learning, and liking, to be subordinate to them and respecting them. This type of game also requires other people with whom to play; people who one depends on for the game to gain life experience and make sense of it; as well as people who are opponents and who one likes to defeat. From the personal point of view, in this game, it is the actions, the decisions and the choices made by each player that count. No one can play for another player. Hence, it is all about controlling procedures, perfecting forms of sharing the development of the game, consenting and respecting, but also knowing how to fight to achieve a good result in every game. In this structure, the institutional aspect and the context, which organize the inter-individual relations, are also very important. At Sabará Children’s Hospital, the older children have access to these types of games; they can find them in the playroom, can challenge the volunteers that visit them in their rooms, or the waiting room, or in the moments preceding a surgery.

### 2.6. Therapeutic Toys

This review will focus on Brazilian research abstracts regarding the use of therapeutic toys. Studies about hospital play can be read in the following articles [16,17,18,19,20,21,22,23,24,25,26].

For Angelo [26], the use of toys in hospitals is one of the resources that makes the child’s experience easier and a valuable information instrument for the healthcare team. In her research, she observed that the therapeutic toy could help the hospitalized child to understand what is going on and what is being done to him or her.

Pinheiro, Lopes and Teixeira [27], in 1993, researched “the influence of toys in the humanization of the assistance of the nursery to the hospitalized child”. Its use is complemented by materials related to the venipuncture therapy, favoring the orientations of the humanization of the assistance to the hospitalized child. Ten children participated in the research, who were evaluated before and after the orientation in the presence and in the absence of their mothers.

Medeiros, Matsumoto and Ribeiro [28] used the institutional therapeutic toy (brinquedo terapêutico institucional (BTI)) to prep relatives and five children of pre-school age who were about to receive intravenous puncture at an emergency room. The BTI was also used to understand the relatives’ perception regarding this preparation. The toy, according to them, allowed the child to: know what to expect and how to participate in the intravenous puncture; understand its purpose; get involved in the situation; and manipulate the material and establish a relation of trust with the professional. Likewise, it allowed the relatives to acknowledge its benefit to the child’s preparation and provide an important source of support and protection. As a conclusion, the authors proposed that the use of the BTI should be integrated with the nursery care provided to children in emergency facilities, assuring humanized, good-quality assistance.

Kiche and Almeida [29] used the therapeutic toy as a strategy of relief for pain and tension during the application of a surgical bandage in children. The objective was to compare the reactions expressed by the child during the application of the bandage before and after the emotional preparation with the institutional therapeutic toy (BTI). Thirty four children admitted for surgery in a public pediatric hospital participated in the research. The children’s behavior and the measuring of pain were considered during the application of the bandage at two moments: before and after the use of the therapeutic toy. The results pointed out that greater adaptation and acceptance of the procedure became more frequent after the toy. The levels of pain also decreased after the use of the therapeutic toy. To them, the therapeutic toy became evident as an effective strategy in reducing the child’s fear, tension and pain during the bandage procedure.

Conceição, Ribeiro, Borba, Ohara and Andrade [30] carried out a research study meant to understand the parents’ perception and the use of the therapeutic toy in prepping the child for intravenous puncture in clinics. The data were collected through semi-structured interviews conducted with eight parents or children’s guardians. The results showed that they approved of this preparation strategy and believed that it benefitted them with respect to knowledge of the procedure, reduced fear, calmed them down and promoted their safety, as well as that of the child, besides providing for a humanized and good quality nursery service for the child and his or her family. The importance of implementing the therapeutic toy in healthcare assistance to children in clinics and basic healthcare facilities is reaffirmed.

Fontes, Mondini, Moraes, Bachega and Maximino [31] carried out an exploratory study about the use of the therapeutic toy as a therapeutic resource in relieving the child’s real and subconscious tensions related to hospitalization. The authors used an observational guideline, applied at two moments: the day before the surgery and the day of the surgery, immediately before its start. They used storytelling and a demonstration of the nurses’ interventions on the toys (dolls) with equipment and materials normally used in hospitalization (gloves, surgical aprons, facial masks and surgical caps). Hence, to them, play favors the interaction of the child in the hospital environment, as well as the expression of the child’s feelings and emotions.

We conclude this analysis of the place of play for the hospitalized child with the study of Weber [32], who researched “the influence of the ludic activity on the child’s anxiety during the pre-surgery period in the clinic’s surgical center”. To evaluate the children’s anxiety, she used the modified Yale Preoperative Anxiety Scale (mYPAS). This scale evaluates five masteries: activities, vocalization, emotional expression, apparent awakening state and interaction with relatives. In each mastery, the evaluator, observing the child’s responses, chooses one of four possibilities, for each mastery, except for vocalization, with six alternatives. Cut-off points are defined, characterizing the positions with or without anxiety. Through this scale, 50 children, five to 12 years old, were evaluated. The children were divided into two groups. One of them, the recreation group, took part in ludic interventions in the playroom room. The other one, the control group, did not receive these interventions. The anxiety scale was applied at two moments: one immediate that is as soon as the children got to the clinic, and the other 15 minutes later. The results showed that, in the second evaluation, the children in the recreation group had their anxiety decreased, and the ones in the control group did not.

## 3. Conclusions

The importance of play was discussed from different points of view. This article considered Brazilian culture and the role of play as a cognitive apparatus, as a healing tool of potentially stressing experiences. There is play that dignifies the child as a human being, who requires specific actions when getting sick. Finally, we presented research about therapeutic play interventions in hospitals and clinics.

Highlighting the recreational, educational, clinical and therapeutic functions of play, its many and important functions were revealed [14]. It creates the basis to develop affectivity and intelligence. Human beings can be affected, but also can creatively affect the world around them. Affection is what affects us, but also what touches us.

At a glance:

− Humanization at the hospital implies values and practice that result from the commitment of all in favor of the well-being and the care of children as subjects with rights;
− Assuming a theoretical vision of the child’s development and the place of play and playful activities is just as important as the practice and the technologies used for the treatment;
− Stress, in the hospital context, is a variable to be considered by everyone, and play is an important way to mitigate it in favor of something more tolerable or even positive;
− There are practices at Sabará Children’s Hospital that benefit the well-being of children, via play and playful activities, during their free time at the hospital;
− There is evidence from research carried out in Brazil that the use of toys benefits the child’s emotional stability during hospitalization.
